# Environmental drivers of bacterial community diversity in the rhizosphere soil of *Zanthoxylum nitidum* (Roxb.) DC from different origins and their correlation with bioactive compounds

**DOI:** 10.3389/fpls.2025.1600456

**Published:** 2025-09-15

**Authors:** Long Chen, MeiRong Huang, Hui Wang, Bilei Huang, Hai Long, Jesus Simal-Gandara, Hua Zhu, Zhonghua Dai, Miao Zhang

**Affiliations:** ^1^ Universidade de Vigo, Nutrition and Bromatology Group, Analytical Chemistry and Food Science, Vigo, Spain; ^2^ Guangxi University of Chinese Medicine, Nanning, Guangxi, China; ^3^ College of Food Science and Technology, Guangdong Ocean university, Guangdong Provincial Key Laboratory of Aquatic Product Processing and Safety, Guangdong Province Engineering Laboratory for Marine Biological Products, Zhanjiang, Guangdong, China; ^4^ Guangxi Key Laboratory of Zhuang and Yao Ethnic Medicine, Guangxi University of Chinese Medicine, Nanning, China

**Keywords:** *Zanthoxylum nitidum* (roxb.) DC, bacteria, rhizosphere soil, high-throughput sequencing, population diversity

## Abstract

**Introduction:**

This study investigated the rhizosphere bacterial communities of *Zanthoxylum nitidum*(Roxb.) DC.

**Methods:**

Using an integrated approach combining 16S rRNA sequencing, redundancy analysis (RDA), and grey relational analysis (GRA) to unravel the correlation among soil environmental factors, microbial diversity, functional potential, and key medicinal compounds.

**Results:**

This study revealed for the first time the effects of environmental factors such as soil pH, organic matter content (ORM), altitude(HT), soil sand(SP) and soil silt (SSG) on the diversity of soil bacterial community, annotated and analyzed the functions of differential flora, and established the correlation between 15 main differential flora (such as *Rudaea, Bradyrhizobium, Gemmatimonas*) and nitidine chloride, which is the main medicinal active component of *Zanthoxylum nitidum*(Roxb.) DC.

**Discussion:**

This study provides theoretical basis and important reference for soil adaptability optimization of *Zanthoxylum nitidum* (Roxb.) DC cultivation from bacterial community regulation, and highlights the supporting value of the study for the sustainable development of medicinal plant resources.

## 1 Introduction

*Zanthoxylum nitidum (Roxb.) DC (Z. nitidum)* is a famous medicinal plant, and its dry root is a Chinese medicine—Liangmianzhen. It has the effects of promoting blood circulation, removing blood stasis, promoting circulation of qi, relieving pain, expelling pathogenic wind, dredging collaterals, removing toxic substances and relieving swelling, and can be used for treating traumatic injury, rheumatic arthralgia, toothache and stomachache. *Z. nitidum* contains many bioactive components, such as alkaloids, flavonoids, volatile oils and polysaccharides (Chen, 2015). Nitidine chloride, one of the primary bioactive constituents of *Z. nitidum*, exhibits multiple remarkable pharmacological properties including anti-inflammatory, analgesic, antimicrobial, and antitumor activities. And nitidine chloride is used as a quality control index for the content determination of *Z. nitidum* in China Pharmacopoeia (Chinese Pharmacopoeia Commission, 2015). *Z. nitidum* is widely distributed in many provinces in southern China, such as Guangxi, Guangdong, and Fujian (Yu, 2007; Guangxi Medical Products Administration, 2008; Tan, 2011).

As a special biological group in rhizosphere soil, rhizosphere soil microorganisms are mainly composed of bacteria, fungi and actinomycetes, which participate in the metabolism of matter and energy between plant roots and soil environment, affecting the physical and chemical properties of rhizosphere soil and the growth of plant roots. Mainly, it can also regulate a variety of medicinal bioactive components in plants. In recent years, researchers have made a series of studies on the rhizosphere microbial biodiversity of medicinal plants, such as ginseng, notoginseng, *Codonopsis pilosula (Franch.) Nannf.* and *Astragalus membranaceus (Fisch.) Bunge*, and their interaction with plants (Guo et al., 2017; Liu et al., 2019).

There are regional differences in quality and composition of *Z.nitidum* (Wu LX. et al., 2023). The formation of this difference is related to climate, soil types and microorganisms (Lu et al., 2006). Soil microorganisms play an important role in these factors. Our previous research found that there were significant differences in rhizosphere soil fungi of *Z.nitidum* in different regions (Zhang et al., 2020). Bacteria is one of the main species of plant rhizosphere microbial population, and its biodiversity is very rich (Peng et al., 2020). Bacteria play a very important role in plants. They form a symbiotic relationship with plants and have a great impact on their physiological functions. They have a variety of regulatory effects on plants, including promoting growth, improving plant stress resistance and affecting the synthesis of secondary metabolites and so on. According to the research of Ni and Xie Qiaoni (Xie, 2016; Ni, 2019) the communities in rhizosphere soil are related to the root growth of medicinal plants and medicinal components synthesis. At present, it was found that there were few reports on the research of rhizosphere soil bacteria of *Z. nitidum.* Therefore, the rhizosphere soils of *Z. nitidum* in different habitats were studied in this paper. Sequencing technique was used to amplify and sequence the V3-V4 variable region of bacterial 16 s rDNA of soil from different habitats. We investigated the diversity and abundance of rhizosphere soil bacterial communities across different production regions and analyzed their associations with environmental factors and nitidine chloride concentrations.

## 2 Materials and methods

### 2.1 Collection of samples

**Figure 1:**
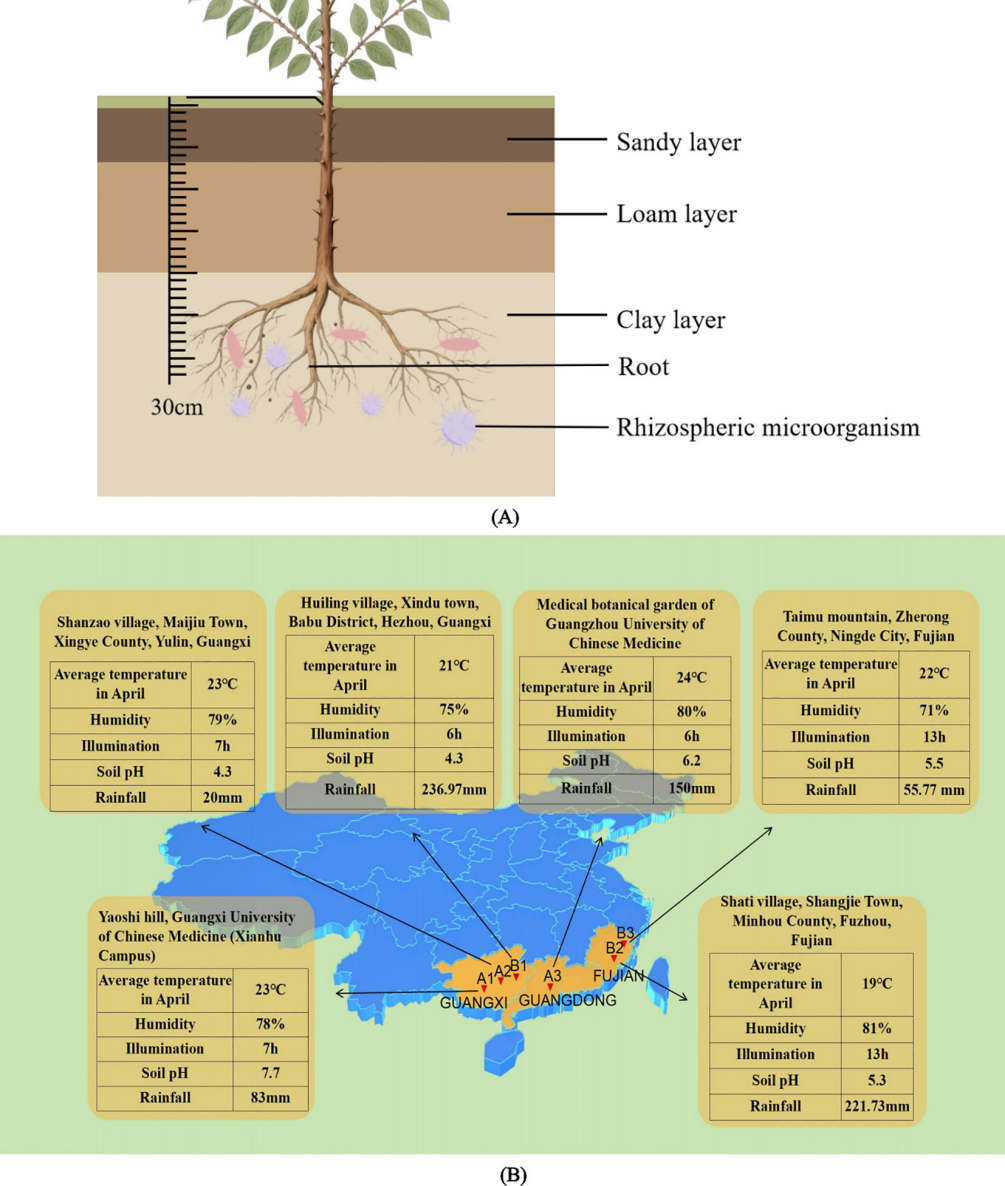
**(A)** Cross-sectional view of soil samples collected in the area around the root of *Z. nitidum* with a depth of 30 cm. **(B)** Ecological infographic of each sampling site. This image reflects the average temperature, humidity, light hours, soil pH, and rainfall of each sampled site (The above data comes from the public information of China Meteorological Data Network).

Based on previous literature research, the sampling locations and methods were determined. The sampling sites were chosen in Guangxi, Guangdong, and Fujian provinces, which are the major producing areas of *Z. nitidum*. Soil samples were collected from an area with a radius of 5 mm and a depth of 30 cm around the roots of *Z. nitidum*, shown in **Figure 1A**. Impurities were removed from the samples before they were thoroughly mixed and placed in ice boxes. Finally, the soil samples were transported back to the laboratory and stored in a refrigerator at 4 °C for future use.

Specifically, sample A1 was obtained from Nanning, Guangxi; sample A2 from Yulin, Guangxi; sample A3 from Guangzhou, Guangdong; sample B1 from Hezhou, Guangxi; sample B2 from Fuzhou, Fujian; and sample B3 from Ningde, Fujian. These soil samples were collected from the root zones of wild plant seedlings. The details of the samples (such as longitude, latitude, height and ecological environment information) are presented in **Table 1** and **Figure 1B**.

**Table 1:** Sample Collection Information.

Sample name	Location	Longitude	Latitude	Height	Time
A1	Yaoshi hill, Guangxi University of Chinese Medicine (Xianhu Campus)	E108°30′3.14″	N22°47′52.85″	96	16 April, 2019
A2	Shanzao village, Maijiu Town, Xingye County, Yulin, Guangxi	E110°04′26.35″	N22°46′14.15″	125	13 April, 2019
A3	Medical botanical garden of Guangzhou University of Chinese Medicine	E113°24′16.37″	N23°03′30.48″	4	6 April, 2019
B1	Huiling village, Xindu town, Babu District, Hezhou, Guangxi	E111°43′52.00″	N24°04′6.60″	56	19April, 2019
B2	Shati village, Shangjie Town, Minhou County, Fuzhou, Fujian	E119°10′19.30″	N26°05′46.12″	24	16 April, 2019
B3	Taimu mountain, Zherong County, Ningde City, Fujian	E119°56′50.32″	N27°11′15.48″	353	20April, 2019

### 2.2 Determination of pH, organic matter content and soil texture in soil samples

According to the agricultural industry standard of China (NY/T 1377 - 2007), the pH value of the soil samples was measured. The organic matter content was determined by referring to the Chinese agricultural industry standard (NY/T 1121.6 - 2006). The soil texture was analyzed using the pipette method specified in the China forestry industry standard (LY/T 1225 - 1999).

### 2.3 Extraction and quality control of soil genomic DNA

DNA was extracted from the soil samples using a soil DNA extraction kit (Mag - Bind Soil DNA kit from OMEGA company). The extraction steps were performed following the kit’s instructions. The concentration of genomic DNA extracted from each sample was determined by Qubit 2.0 and loaded on 1% agarose gel to detect DNA integrity. Sample DNA meeting the requirements of concentration (0.05 ng/ul~120 ng/ul) and integrity was amplified by PCR.

### 2.4 Bacterial V3~V4 region amplification and sequencing

Bacterial 16S rDNA Amplification and Sequencing: the V3 - V4 variable region of bacterial 16S rDNA was chosen as the amplification target region. Illumina MiSeq paired - end sequencing (2 × 300 bp) was employed to perform the sequencing. PCR Primers: the primers utilized in the PCR incorporated the V3 - V4 universal primers specific to the sequencing platform. The 341F primer sequence was 5’-CCTACGGGNGGCWGCAG-3’, and the 805R primer sequence was 5’-GACTACHVGGGTATCTAATCC-3’. First - Round PCR Amplification: the reaction system for the first - cycle PCR amplification was as follows: 15 μl of 2×Taq master Mix, with Bar - PCR primer F (10 μM) and Primer R (10 μM) added to a total volume of 30 μl. The PCR amplification program consisted of the following steps: initial denaturation at 94 °C for 3 min, five cycles of: 94 °C for 30 s, 45 °C for 20 s, and 65 °C for 30 s, twenty cycles of: 94 °C for 20 s, 55 °C for 20 s, and 72 °C for 30 s, final extension at 72 °C for 7 min. Second - Round PCR Amplification: in the second round of PCR amplification, primers compatible with Illumina bridge PCR were introduced. The amplification reaction system contained 15 μl of 2×Taq master Mix, 1μl each of primer F (10 μM) and Primer R (10 μM), and 2 μl of the PCR products from the previous round, with the total volume adjusted to 30 μl. The PCR amplification program was as follows: initial denaturation at 95 °C for 3 min, thirty cycles of: 94 °C for 20 s and 72 °C for 30 s, final extension at 72 °C for 5 min. PCR products were detected by agarose electrophoresis. The amplification products that meet the requirements of more than 400bp are recovered by magnetic bead method. The recovered DNA was accurately quantified by using Qubit 3.0 DNA detection kit, and was mixed in equal amount of 1: 1 for sequencing. When mixed equally, the amount of DNA in each sample was 10ng, and the final sequencing concentration was 20 pmol. Finally, the samples were sent to Sangon Biotech (Shanghai) Co., Ltd. for sequencing.

### 2.5 Data analysis

(1) Processing and statistical analysis of soil physical and chemical data: Excel 2013 was used for data processing and statistical analysis, which was listed in the format of “mean ± standard deviation”. (2) Processing and statistical analysis of Miseq sequencing sequence data: After the sequencing data was stripped of primer joints, the sequences were spliced by PEAR (version: 0.9.6), and each sample data was identified and cut by PRINSEQ software (version: 0.20.4) according to the tag sequence. Then, the low-quality data in each sample data was filtered through quality control to obtain qualified sequences for each sample (see quality control data chart, which was placed in **Supplementary Material**). After chimeras and nonspecific amplified sequences were removed from the quality-controlled data using USEARCH (version: 5.2.236) and UCHIME (version: 4.2.40) software, the effective sequences of each sample were obtained. Using a similarity threshold of 97%, the valid sequences of each sample were clustered into operational classification units (OTU), and the analysis, screening, and classification of OTUs were completed using USEARCH software (version: 5.2.236). RDP classifier software was used to classify species, with the confidence threshold set to 0.8. The OTU sequences were compared with the 16S bacterial database in RDP (https://sourceforge.net/projects/rdp-classifier/), and sequences with similarity > 90% and coverage > 90% were set to be used for subsequent classification, while sequences that did not meet the conditions were classified as unclassified. R software (version: 3.2) was used to complete the analysis and mapping of α and β diversity of samples, the function of differential flora, and the influence of soil environmental factors on flora structure. GenBank’s functional gene database was used for functional analysis of different flora.

## 3 Results

### 3.1 Variable soil pH and organic matter, nearly uniform texture

The results of the soil pH determination for six samples are presented in **Table 2**. Among these samples, all except A1 (pH = 7.7) were acidic (pH < 7). The pH values of the six samples followed the order: A1 > A3 > B3 > B2 > A2 = B1. In terms of organic matter content, sample A1 (54.4 ± 0.02 g/kg)exhibited the highest value among all the samples, while sample B3 (9.55 ± 0.25 g/kg) had the lowest. Regarding the clay particle level, sample A1 (593.11 ± 0.03 g/kg) had the highest content, whereas sample A3 (278.05 ± 22.96 g/kg) had the lowest. Conversely, sample A3 (523.05 ± 42.73 g/kg) had the highest content of the grain level, and sample A1 (192.84 ± 3.79 g/kg) had the lowest. For the powder (sand) grain level, sample B2 (221.58 ± 0.38 g/kg) had the highest content, and sample A3 (198.9 ± 16.29 g/kg) had the lowest. In terms of soil texture, all samples except A3, which was classified as sandy clay loam, were identified as clay.

**Table 2:** The results of PH、organic matter content and soil texture of the soil samples.

Sample name	Ph value	Organic matter content	Clay content (<0.002 mm)	Sand content(2.0-0.05mm)	Silt content(0.05-0.002mm)	Soil texture
		(g/kg)	g/kg	g/kg	g/kg	
A1	7.7 ± 0.1	54.45 ± 0.02	593.11 ± 0.03	192.84 ± 3.79	214.05 ± 4.19	clay
A2	4.3 ± 0.1	35.48 ± 0.04	462.05 ± 0.01	318.13 ± 0.01	219.83 ± 0.06	clay
A3	6.2 ± 0.5	33.54 ± 2.37	278.05 ± 22.96	523.05 ± 42.73	198.90 ± 16.29	sandy clay loam
B1	4.3 ± 0.3	39.52 ± 1.95	492.00 ± 25.67	298.59 ± 15.71	209.41 ± 11.04	clay
B2	5.3 ± 0.0	33.98 ± 0.02	424.69 ± 0.02	353.74 ± 0.01	221.58 ± 0.38	clay
B3	5.5 ± 0.1	9.55 ± 0.25	443.08 ± 11.22	356.83 ± 9.55	200.10 ± 5.07	clay

### 3.2 Sequencing results

Through high - throughput sequencing analysis of the V3 - V4 variable region of bacterial 16S rDNA from six rhizosphere soil samples, a total of 511,904 original readings were obtained. Subsequently, after merging the double - terminal reads and performing quality control filtration, 499,430 clean tags were acquired. The ARCH software (version 5.2.236) was employed to eliminate some non -amplified sequences in all samples and correct sequencing errors. Chimeras were identified using the Uchime software (version 5.2.236). Then, the sequences with chimeras removed were compared with the representative sequences in the database using BLASTN to detect and remove out - of - target sequences. Finally, 484,452 effective sequences were successfully obtained. The processing results of the sequencing data for each sample were presented in **Table 3**.

**Table 3:** Statistical tables of the results of sequencing data processing for samples.

Sample name	Number of raw reads	Number of reads remaining after QC	Number of organelle tissue sequences matched	Number of out-target region sequences	Number of chimeras	Number of final effective reads
A1	79142	77682	1033	0	5328	71321
A2	99113	95984	221	2	774	94987
A3	129644	127367	98	0	3933	123336
B1	68191	66204	46	2	636	65520
B2	69206	67436	1385	1	472	65578
B3	66608	64757	245	1	801	63710

### 3.3 Alpha diversity analysis

Alpha diversity analysis reflected the richness and diversity of fungal communities in rhizosphere soil samples of *Z. nitidum* from different habitats. In the context of α-diversity indices, the Chao 1 index and the ACE index were indicative of the richness of the sample community, while the Shannon index and the Simpson index reflected community diversity. A higher Shannon index implied greater biodiversity in the samples, whereas a higher Simpson index suggested lower biodiversity. The coverage index indicated the extent to which the sequencing results represented the actual situation of the samples (Cai et al., 2018; Fu et al., 2019). As shown in **Table 4**, Chao 1 index and ACE index of six soil samples were ranked as follows: A3 > B3 > A2 > B2 > A1 > B1. Both indices demonstrated the same trend in soil fungal abundance: sample A3 had the highest abundance, followed by B3, and B1 had the lowest. Besides, the Shannon index and the Simpson index of the six soil samples showed the same order in terms of sample diversity: A3 > A1 > A2 > B1 > B3 > B1. To sum up, it showed that the bacteria in the six soil samples all had high species richness and high similarity, but there were some differences in their community structure and relative abundance of fungal groups. The bacteria in sample A3 had the highest species diversity, followed by sample A1, and sample B1 had the lowest species diversity.

**Table 4:** Analysis of α diversity index of bacterial community in samples.

Sample name	Seq_num	Shannon_index	ACE_index	Chao1_index	Coverage	Simpson_index
A1	68509	6.58982	4828.617912	4651.211268	0.983958	0.003667
A2	91460	6.123974	5111.996477	4893.09205	0.986803	0.006909
A3	118559	6.668153	7554.056437	7369.780325	0.986091	0.008215
B1	61514	6.10682	4629.651244	4424.911252	0.98163	0.007958
B2	61750	5.684475	4929.562798	4724.361072	0.980551	0.036926
B3	59787	5.807217	5144.345854	4914.99866	0.978691	0.029716

The coverage rate of each sample exceeded 0.97, suggesting that the sequencing results accurately represented the actual bacterial composition in the samples.

### 3.4 Analysis of flora difference

**Figure 2:**
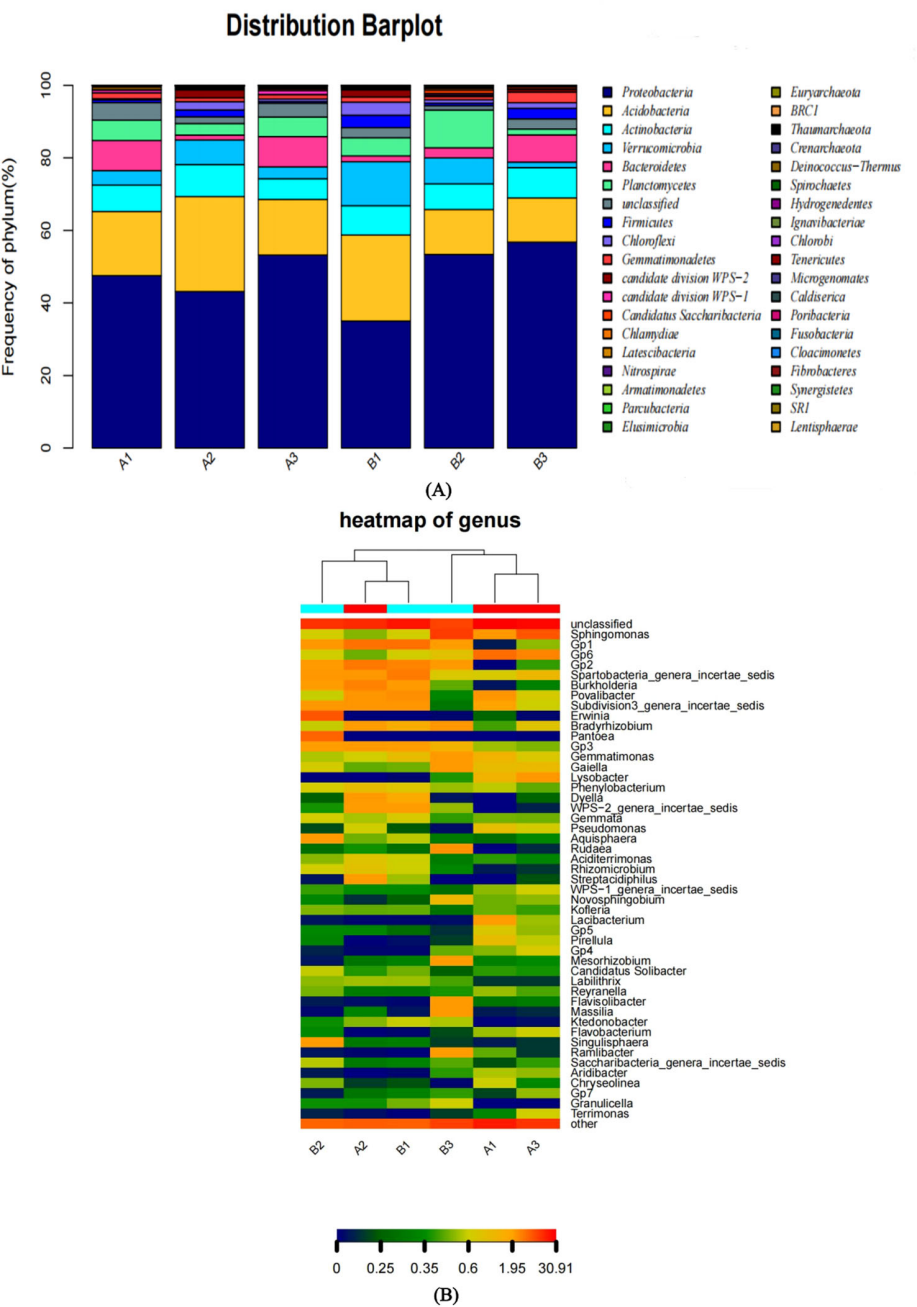
**(A)** Bacterial community structure of various samples at the Phylum classification level. Different colors represent different Phylum; different color block widths represent relative abundance ratios of different species. **(B)** Abundance heat map of bacteria genues in samples. Each column in the graph represents a sample, rows represent the structure of the group. The color block of heat map represents the relative abundance of species. The redder the color block is, the higher the relative abundance. On the contrary, the more blue the color is, the lower the relative abundance. At the same time, heat map analysis can also show the cluster analysis of samples.

The optimal comparison results for the OTU sequences were then selected. The classification results of six bacterial community samples at different taxonomic levels were presented in **Table 5**. At the phylum classification level, the composition of the bacterial communities in the six soil samples was shown in **Figure 2A**. The data from **Table 5** and **Figure 2A** indicated that the bacteria in the samples primarily belonged to ten bacterial phyla, namely *Proteobacteria, Acidobacteria, Actinobacteria, Verrucomicrobia, Bacteroidetes, Planctomycetes, Firmicutes, Chloroflexi, Gemmatimonadetes*, and *Unclassified. Notably, there were significant differences in the composition and* sp*ecies richness of the bacterial* communities across all samples. *Proteobacteria* and *Acidobacteria* emerged as the dominant bacteria in the bacterial communities (> 10% relative abundance). Among them, *Proteobacteria* showed the most prominent dominance and the highest species richness. For instance, in sample B3, the abundance of *Proteobacteria* was the highest, reaching 56.77%, followed by sample B2 with 53.39%, and sample B1 with the lowest abundance at 35.03%.

**Table 5:** The classification results of 6 samples of the bacterial flora at various classification levels.

Type name	Classification level	Sample					
		A1	A2	A3	B1	B2	B3
Colony number	Phylum	26	26	25	25	24	22
	Class	62	59	65	58	59	56
	Order	92	91	102	88	93	89
	Family	167	157	178	147	163	158
	Genus	372	293	404	300	324	374

In a heat map (**Figure 2B**), each color block represented the relative richness of a species. A redder color block indicated a higher relative abundance, while a bluer color signified a lower relative abundance. Simultaneously, heat map analysis could also present the results of sample clustering (Wang et al., 2018; Qin et al., 2019). Based on the heat map clustering trees at each taxonomic level, when the similarity of community structure and relative species abundance of samples was used as the criterion for clustering analysis, the clustering results of the six samples were consistent across all taxonomic levels. The six samples could be roughly divided into two groups. Samples A2, B1, and B2 were classified into one group, while samples A1, A3, and B3 were placed in another. Within the group of A2, B1, and B2, samples A2 and B1 exhibited a high degree of similarity in community structure and relative species richness. Similarly, within the group of A1, A3, and B3, samples A1 and A3 showed a high level of similarity in community structure and relative species richness.

However, there were certain disparities in the relative species richness. Excluding the *Unclassified* group, the top 5 genera in terms of relative abundance for each sample were as follows: For sample A1, the top 5 genera and their relative abundances were: *Unclassified* at 30.91%, *GP6* at 10.74%, *povalicharacter* at 3.52%, *Sphingomonas* at 3.41%, and *Lacibacterium* at 2.02%. For sample A2, they were: *Unclassified* at 22.6%, Gp1 at 9.52%, Gp2 at 8.93%, *Burkholderia* at 7.36%, and *Subdivision3_genera_incertae_sedis* at 3.48%. For sample A3, the values were: *Unclassified* at 28.84%, *Sphingomonas* at 14.73%, *Gp6* at 6.66%, *Lysobacter* at 3.27%, and *Spartobacteria_genera_incertae_sedis* at 1.56%. For sample B1, the top 5 genera had relative abundances of: *Unclassified* at 25.99%, *Gp1* at 10.28%, *Spartobacteria_genera_incertae_sedis* at 8.2%, *Gp2* at 6.64%, and *Povallibacter* at 4.56%. For sample B2, the relative abundances were: *Unclassified* at 20.95%, *Erwinia* at 14.72%, *Pantoea* at 12.2%, *Subdivision3_genera_incertae_sedis* at 3.4%, and *Gp1* at 3.18%. For sample B3, the top 5 genera and their abundances were: *Sphingomonas* at 19.14%, *Unclassified* at 18.32%, *Rudaea* at 4.04%, *Bradyrhizobium* at 3.07%, and *Gemmatimonas* at 2.93%.

### 3.5 Beta diversity analysis

**Figure 3:**
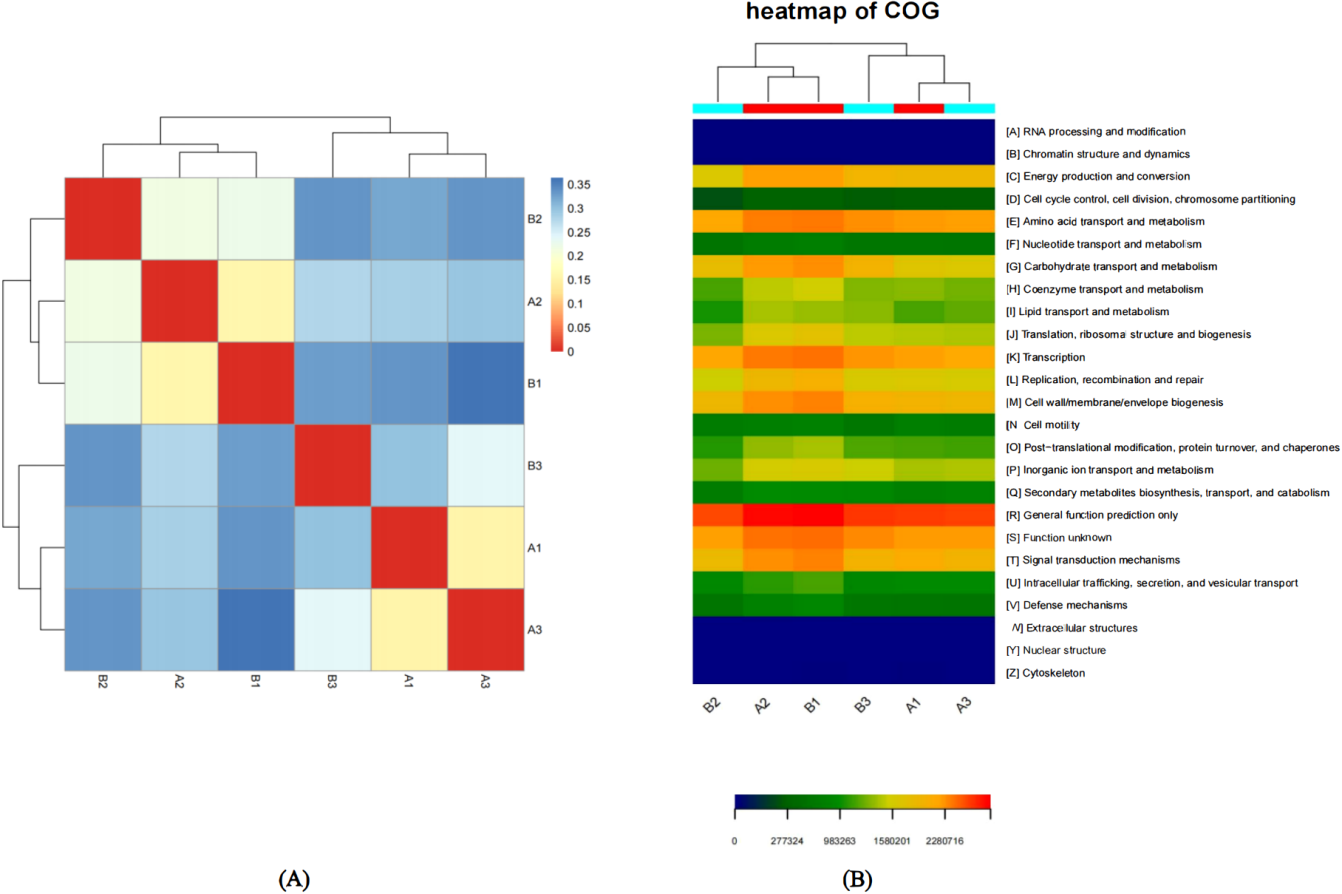
**(A)** Sample distance heat map.The color block represents the distance value. The redder the color, the closer the distance between samples, the higher the similarity, and the bluer the distance. The samples are clustered in the heat map, and the distance relationship between samples can also be seen through the cluster tree. **(B)** Functional Abundance Heat Map based on COG. Functional Abundance Heat Map is drawn by functional abundance matrix. Each column in the map represents a sample, and the row represents the function, and the color block represents the functional abundance value. The redder the color, the higher the abundance, and the bluer the color. In addition, the samples are clustered by the heat map. The more similar the distribution of bacterial flora in the samples, the closer the samples are and the closer they are in the cluster tree above the map. There is a color block at the top of the graph, and the samples from the same group have the same color.

Beta diversity was the comparison of diversity between different ecosystems, and it was the rate of change of species composition along the environmental gradient or between communities, which was used to express the response of biological species to environmental heterogeneity. Distance thermogram could intuitively show the distance relationship between samples by color, that is, the similarity between samples. The color block represented the distance value. The redder the color, the closer the distance between samples, the higher the similarity, and the bluer the distance. The samples were clustered in the heat map, and the distance relationship between samples could also be seen through the cluster tree. Using the heat map package of R, a heat map was drawn according to the Unifrac distance matrix between samples. As shown in **Figure 3A**, samples A1 and A3 were relatively close, indicating a relatively high similarity, so they could be classified into one category. Similarly, samples A1 and B2 could also be grouped together. The distance between other samples was relatively large, indicating that the similarity was relatively low, and each of these samples could be regarded as a different category. Among them, the high similarity between samples A1 and A3 might be attributed to their similar latitudes, while the high similarity between samples A2 and B1 might be attributed to their close geographical locations. Therefore, we speculated that geographical location (such as longitude or latitude) may have had an influence on the OTU abundance of samples.

### 3.6 Analysis of functional abundance of bacterial flora by heat map

**Figure 4:**
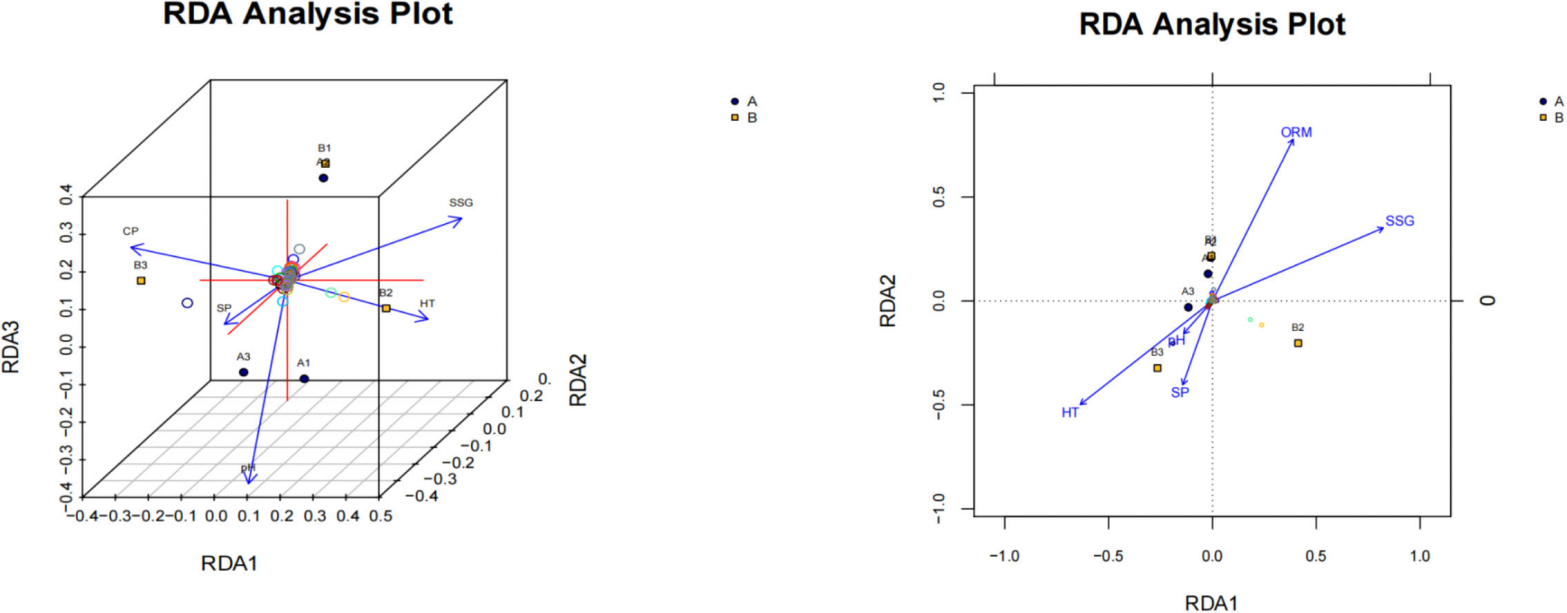
RDA analysis of the relationship between samples and environmental factors. The arrows in the figure represent the relative positions of different environmental factors in the plane, respectively. The longer the arrows, the greater the effect. The relationship between environmental factors and bacterial flora structure was estimated according to the angle between the arrow and the sample center connection in **Figure 5**. When the angle was sharp, it showed a positive correlation between them. When obtuse angle was observed, it showed a negative correlation between them. Projection through the sample to the connecting line or extension line of each environmental factor arrow, and the closer the projection point to the arrow, the greater the influence of the environmental factors on the bacterial community structure (Lai and Mi, 2010).

Heat map could reflect the abundance information of flora function by color change, and could intuitively express the functional abundance value by defined color depth. At the same time, six samples and functional information were clustered and rearranged, and the results after clustering were shown in **Figure 3B**. Based on the COG functional classification abundances, the functional distribution of microbial communities in six samples was analyzed across 25 functions, such as General function prediction only, Amino acid transport and metabolism, Energy production and conversion, Transcription, Cell wall/membrane/envelope biogenesis, and so on. The sequences of the functions of the microbial flora in the six samples were compared to predict and analyze the differences in their abundances. **Figure 4** showed the functional abundances of the microbial communities in the six samples. We could clearly and intuitively see that the highest functional abundance of the six samples was General function prediction only, and the lowest functional abundance was RNA processing and modification, Chromatin structure and dynamics, Extracellular structures, Nuclear structure, Cytoskeleton. In addition, on the Heat map, we could clearly see that the flora functions of sample A2 and sample B1 were highly similar. The highest abundance of flora functions of sample A2 and sample B1 was General function prediction only, followed by Function unknown, Transcription, Signal transduction mechanisms, Amino acid transport and metabolism, Carbohydrate transport and metabolism. Similarly, the flora functions of sample A1 and sample A3 were very similar. The highest abundance of flora functions in sample A2 and sample B1 was General function prediction only, followed by Function unknown, Amino acid transport and metabolism, Transcription. In summary, the samples with close geographical distance often had high similarity in the cluster analysis of six samples, and their microbial community functions were also closely related. This observation show the potential correlation between geographical location and the functional characteristics of microbial communities in these samples.

### 3.7 RDA analysis of soil bacterial flora and environmental factors

According to DCA analysis, the axis length was less than 3.0. Therefore, Redundancy Analysis (RDA) was employed to analyze the correlation between soil bacterial flora and environmental factors. In this study, soil pH, organic matter content (ORM), altitude (HT), soil clay (CP), soil sand (SP), and soil silt (SSG) were considered as environmental factors. RDA analysis was conducted in combination with the sample clustering results, and the findings are presented in **Figure 4**. As depicted in **Figure 4**, the central line of sample A1 formed an acute angle with the vectors representing soil silt content and organic matter content, while it formed an obtuse angle with those representing pH value, altitude, and soil sand content. These results indicate that the community structure at the sampling site of sample A1 was positively correlated with soil silt (SSG) and organic matter (ORM) content, and negatively correlated with pH, elevation (HT), and soil sand (SP). The patterns observed for samples A2, B1, and B2 were consistent with that of sample A1. In contrast, the patterns for samples A3 and B3 were opposite to those of the aforementioned samples.

By measuring the distance from the projection point of the A1 sample on the arrow line or its extension line of various environmental factors to the arrow, we can assess the influence of these environmental factors on the bacterial community structure of the A1 sample. The order of influence is as follows: pH > soil sand (SP) > altitude (HT) ≈ soil silt (SSG) ≈ organic matter content (ORM).

For the A2 sample, the impact on its bacterial community structure is ranked as: pH > soil sand (SP) > organic matter content (ORM) > altitude (HT) ≈ soil silt (SSG). The influence on the bacterial community structures of samples A3 and B1 is consistent with that of the A1 sample. Regarding the B2 sample, the effect on its microbial community structure is ranked as: pH > soil sand (SP) > soil silt (SSG) > altitude (HT) > organic matter content (ORM). For the B3 sample, the order of influence on its microbial community structure is: soil sand (SP) > pH > altitude (HT) > soil silt (SSG) ≈ organic matter content (ORM) (Jiang and Song, 2010).

### 3.8 Analysis of the relationship between microorganisms

**Figure 5:**
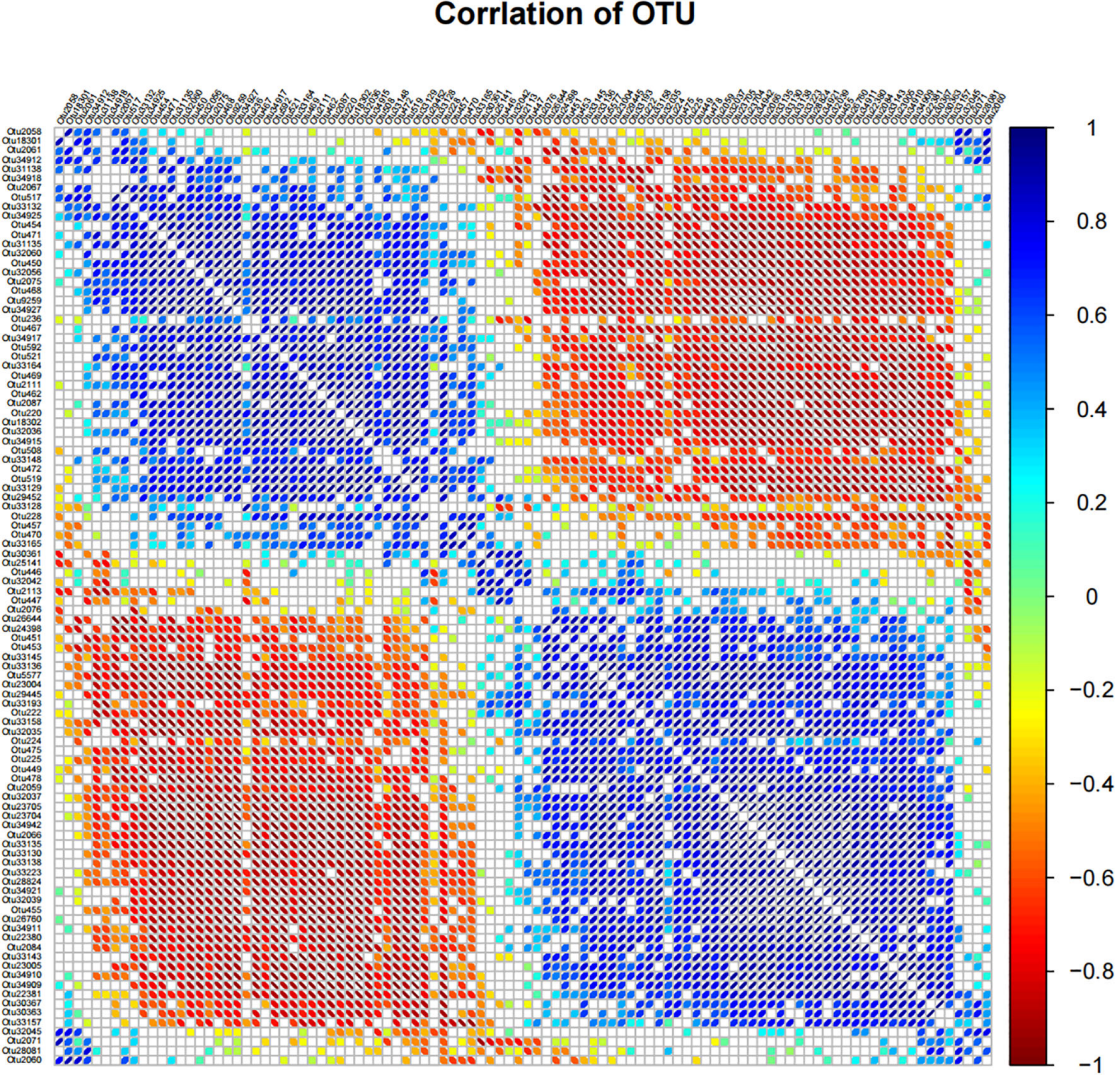
Correlation matrix diagram based on OTU. In the figure, the size of the ellipse represents the absolute value of the correlation coefficient (the larger the absolute value of the correlation, the smaller the ellipse), the left oblique is positively correlated, the right oblique is negatively correlated, and the color changes with the right color scale. Only results with p-values less than 0.05 are shown in the figure.

Correlation analysis is a classical method to test the interaction between microorganisms. In this study, we aim to determine the significant correlation, strong correlation, positive correlation and negative correlation in the microbial community of six samples. In the correlation matrix diagram based on OTU, the size of the ellipse in the figure represented the absolute value of the correlation coefficient (the greater the absolute value of the correlation, the smaller the ellipse), the left oblique was positively correlated, the right oblique was negatively correlated, and the color changed with the right color scale. For analysis, we selected the top 100 most abundant OTU information and conducted bilateral tests. Only the results with p value less than 0.05 are shown in the figure. The results are shown in **Figure 5**. Significant negative correlations were observed between the following pairs of OTUs: Otu33165 and Otu228, Otu472 and Otu14485, Otu472 and Otu33129, Otu462 and Otu2087, Otu18302 and Otu2087, Otu32039 and Otu28824. Negative correlations were detected between: Otu33148 and Otu33165, Otu2087 and Otu556, Otu2075 and Otu469, Otu2075 and Otu33148, Otu34911 and Otu33143, Otu447 and Otu33223. Significant positive correlations were found in the following pairings: Otu14485 and Otu33157, Otu519 and Otu34911, Otu2087 and Otu23005, Otu2087 and Otu34910, Otu33157 and Otu33165, Otu29445 and Otu31135. Positive correlations were identified between: Otu29452 and Otu30363, Otu14485 and Otu2126, Otu2111 and Otu2126, Otu33223 and Otu220, Otu2059 and Otu508, Otu2113 and Otu2067.

### 3.9 Grey correlation analysis of soil bacterial community abundance and nitidine chloride content

Z.*nitidum* is rich in a diverse array of active ingredients, such as alkaloids, flavonoids, volatile oils, and polysaccharides. These components endow *Z. nitidum* with a wide spectrum of pharmacological effects. Among the alkaloids present in *Z. nitidum*, nitidine chloride is particularly well - known. It exhibits anti - tumor, analgesic, anti - inflammatory, and cardiovascular protective properties, which are emblematic of the remarkable medicinal value of *Z. nitidum*.

**Figure 6:**
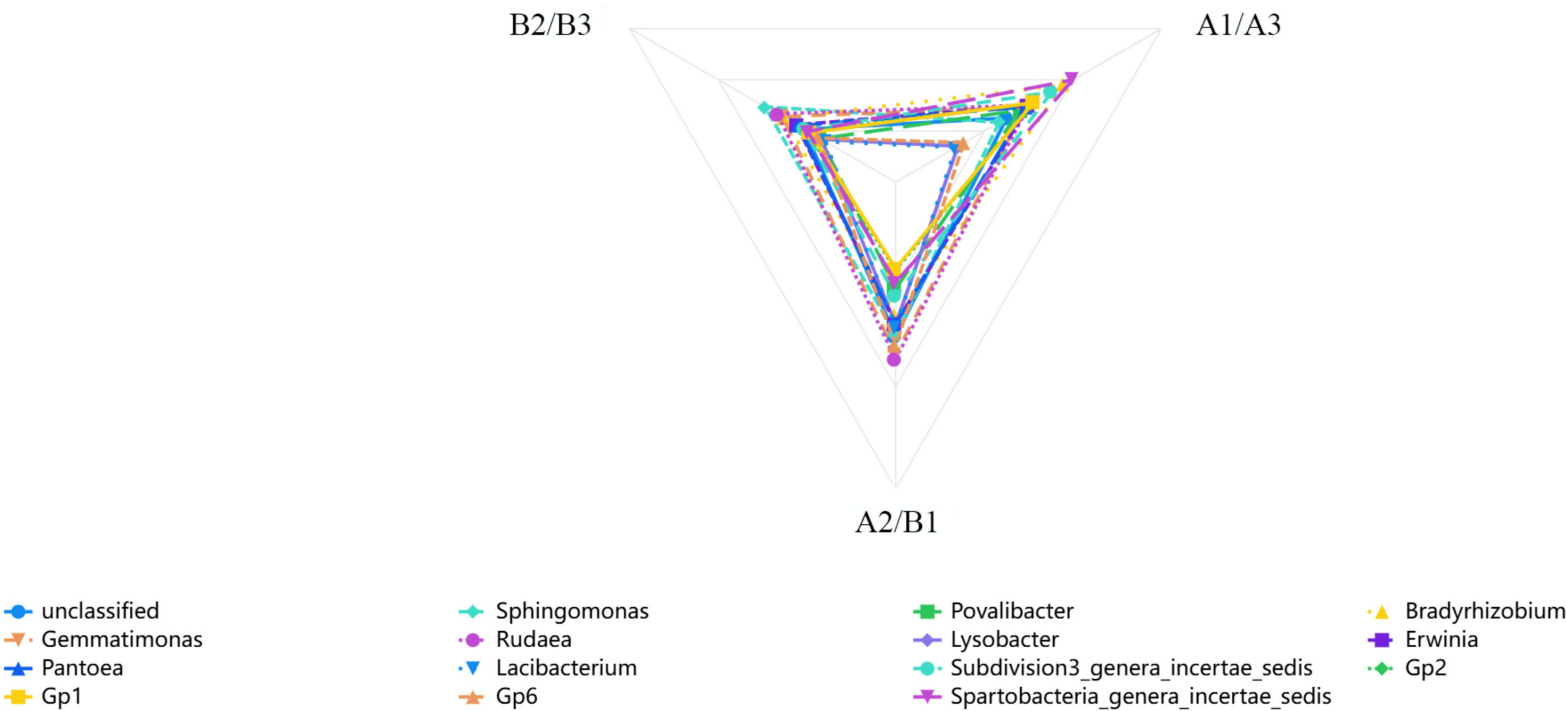
The correlation coefficient between the abundance of soil flora and the content of nitidine chloride in *Z. nitidum* from different producing areas (different colors represent different soil flora, and the closer the flora is to the top, the stronger the correlation between the abundance of soil flora and the content of nitidine chloride in *Z. nitidum* from different producing areas).

Cluster analysis of heat map based on 6 samples (**Figure 3A**), a grey correlation analysis was conducted between the soil bacterial community abundance and nitidine chloride content. Fifteen bacterial taxa, including *unclassified* bacteria, *Sphingomonas*, *Povallibacter*, *Rudaea*, *Bradyrhizobium*, and *Gemmatimonas*, were selected as characteristic sequences, while nitidine chloride served as the parent sequence. A resolution coefficient of 0.50 was employed. The correlation values ranged from 0 to 1, with higher values indicating a stronger correlation with nitidine chloride. The data regarding the soil flora abundance and nitidine chloride content of *Z. nitidum* from different habitats are presented in **Table 6**. The results of the correlation coefficients are shown in **Table 7**, and the correlation degrees are presented in **Table 8**. As can be observed from **Table 7**, **Table 8**, and **Figure 6**, among the 15 soil bacterial taxa and nitidine chloride content, *Rudaea* had the highest comprehensive evaluation, with a correlation degree of 0.762. It was followed by *Bradyrhizobium* (correlation degree: 0.744), *Gemmatimonas* (correlation degree: 0.739), *Sphingomonas* (correlation degree: 0.690), and *Erwinia* (correlation degree: 0.668). In contrast, *Lacibacterium* had the lowest correlation degree, at 0.481.

**Table 6:** The abundance value of soil microbiota and the content of chlorinated two-sided needle alkali in different origins.

Sample name	*unclassified*	*Sphingomonas*	*Gp6*	*Gp1*	*Spartobacteria_genera_incertae_sedis*	*Gp2*	*Erwinia*	*Povallibacter*	*Subdivision3_genera_incertae_sedis*	*Bradyrhizobium*	*Lacibacterium*	*Lysobacter*	*Pantoea*	*Rudaea*	*Gemmatimonas*	Content of nitidine chloride
A1/A3	29.875%	9.07%	8.7%	0.405%	1.355%	0.24%	0.095%	2.34%	1.485%	0.91%	1.42%	2.445%	0.005%	0.035%	1.41%	0.205% (Qin et al., 2006; Tan et al., 2011)
A2/B1	24.295%	0.88%	0.845%	9.9%	5.66%	7.785%	0.01%	4.005%	3.58%	2.055%	0.02%	0.01%	0.005%	0.29%	1.245%	0.25% (Qin et al., 2006; Wu L. et al., 2023)
B2/B3	19.635%	10.105%	1.17%	2.75%	2.11%	2.55%	7.37%	0.655%	1.815%	2.025%	0.035%	0.215%	6.1%	2.12%	1.915%	0.87% (He et al., 2024)

**Table 7:** The results of correlation coefficient between the abundance of soil flora and the content of nitidine chloride in different producing areas.

Sample name	*Unclassified*	*Sphingomonas*	*Povallibacter*	*Bradyrhizobium*	*Gemmatimonas*	*Rudaea*	*Lysobacter*	*Erwinia*	*Pantoea*	*Lacibacterium*	*Subdivision3_genera_incertae_sedis*	*Gp2*	*Gp1*	*Gp6*	*Spartobacteria_genera_incertae_sedis*
A1/A3	0.627	0.585	0.703	0.950	0.735	0.754	0.352	0.751	0.735	0.338	0.882	0.765	0.777	0.387	1.000
A2/B1	0.753	0.747	0.521	0.654	0.841	0.865	0.697	0.694	0.693	0.708	0.558	0.431	0.421	0.798	0.491
B2/B3	0.516	0.737	0.424	0.626	0.639	0.668	0.419	0.559	0.550	0.396	0.515	0.501	0.483	0.431	0.495

**Table 8:** The correlation between the abundance of soil flora and the content of nitidine chloride in different producing areas.

Colony of bacteria	Degree of association
*Rudaea*	0.762
*Bradyrhizobium*	0.744
*Gemmatimonas*	0.739
*Sphingomonas*	0.69
*Erwinia*	0.668
*Spartobacteria_genera_incertae_sedis*	0.662
*Pantoea*	0.659
*Subdivision3_genera_incertae_sedis*	0.651
*unclassified*	0.632
*Gp2*	0.566
*Gp1*	0.56
*Povallibacter*	0.549
*Gp6*	0.539
*Lysobacter*	0.489
*Lacibacterium*	0.481

Therefore, it can be inferred that in *Z. nitidum* soils from different habitats, a higher abundance of *Rudaea*, *Bradyrhizobium*, and *Gemmatimonas* may be associated with a higher nitidine chloride content in *Z. nitidum*.

## 4 Discussion

In the present study, 6 medicinal plant rhizosphere soils collected from Guangxi, Guangdong and Fujian, three provinces in southern China, were used to study the diversity of bacterial flora in different soil samples using high throughput sequencing technology. The results showed that the bacterial flora in the soil samples of 6 habitats had high biodiversity, but the flora structure and species richness were significantly different. From the taxonomic point of view, the bacteria in the six soil samples mainly come from nine bacterial phyla, such as *proteobacteria, acidobacteria, actinobacteria, verrucomicrobia,bacteroidetes, planctomycetes, firmicutes, chloroflexi* and *gemmatimonadetes*. A small amount of bacteria come from unclassified species. Among them, the flora of bacteria is the highest, especially *Proteobacteria* and *Acidobacteria*, and the proportion of bacteria from other phyla in each sample was different. These variations in the dominant taxa may be associated with differences in the ecological environments and soil nutrient compositions of the samples. The average proportion of *proteobacteria* and *acidobacteriain* the soil bacterial population reached 48.18% and 17.89% respectively, and they also had an absolute advantage in the bacterial population of all sample. So they were the dominant bacteria in each sample. At the genus classification level, the bacterial populations in each soil sample had similarities, but there were large differences overall. The difference in dominant bacterial genus was also very obvious. Except for unclassified bacteria, the species and quantity of dominant bacteria in each sample are different. Thus, the difference in the rhizosphere microbial environment of *Z.nitidum* from different habitats was reflected. Rhizosphere microorganisms could regulate plant growth and energy metabolism, such as promoting the development of plant roots system and the intake and distribution of nutrients (Jiang and Song, 2010). Therefore, based on the difference of rhizosphere microorganisms, the effects of rhizosphere microorganisms on plant growth and energy metabolism are studied, and the differences of the same plants in different habitats are explored, which provides an idea for revealing the formation mechanism of authentic medicinal materials (Jiang et al., 2009).

In the micro-ecosystem of “plant-soil-microorganism”, soil is the main place for energy exchange and metabolism of plants and rhizosphere microorganisms. At the same time, it is an important carrier and link for the three to play an interactive role in (Lambers et al., 2009; Mai et al., 2017). The results of the present study showed that there was a close relationship between bacterial populations in the rhizosphere soil of Z. nitidum and environmental factors such as soil pH, soil organic matter content, altitude, soil sand and soil silt. According to the research results of Zhu Jinghua (Zhu et al., 1994) and Deng Tingfei (Deng et al., 2014), the differences in the number of soil bacteria might be caused by the differences in soil nutrients in different regions. Soil nutrients are closely related to the soil mechanical composition, so the mechanical composition of rhizosphere soil will also affect the bacterial population structure of rhizosphere soil, resulting in differences in bacterial population diversity in the rhizosphere soil of the same plant in different regions (Mao et al., 2019; Wang et al., 2019). In addition, plant growth also has an impact on the structure of bacterial population and soil structure. From the analysis of the influence of environmental factors on the bacterial population structure of soil samples, it can be seen that soil PH has the greatest influence on the bacterial population structure of *Z. nitidum* rhizosphere soil, followed by soil sand, and the other environmental factors had little influence on the bacterial population structure. Combined with the information collected from soil sample, the physical and chemical properties and mechanical composition of soil, the analysis result are consistent with the geographical distribution of *Z. nitidum* resources and its growing soil environment. From the species composition of the bacterial population, there were a number of common and unique bacterial species in the rhizosphere soil of *Z. nitidum* from different habitats (shown in **Figure 2C**), and there were significant differences in the abundance of the bacterial species. The difference of possible species is related to the difference of plant genotype or ecotype, and the difference of species abundance is related to the ecological condition of habitats.

Soil factors in the soil first affect the absorption function and signal transmission process of plant roots, while nutrients absorbed by roots and perceived environmental signals will be further transmitted to plants, affecting the synthesis and activity regulation of enzymes. The change of enzyme activity and the dynamic balance of metabolic pathways jointly determine the synthesis, accumulation and transformation efficiency of effective components in medicinal plants. But the soil structure is similar. Only from the aspect of soil factors, the factors that affect the different contents of secondary metabolites of plants from different habitats may come from rhizosphere soil microorganisms, and the direct effect of microorganisms on the accumulation of secondary metabolites of plants is to directly synthesize effective components. For example, according to the research of Jianmu Su (Su et al., 2023), *Streptomyces Strep-4* in soil can independently synthesize monoterpene precursors in the rhizosphere of citrus sinensis, and directly enter the roots to enhance the contents of active components such as limonene and γ-terpinene. The indirect effects of microorganisms on the accumulation of plant secondary metabolites include regulating soil energy, nutrient transformation and cofactor supply (Su et al., 2023) and gene expression regulation. For example, Zeng M (Zeng et al., 2018) found that *Burkholderia* sp. upregulated the indigo synthesis gene BIA1/BIA2 in the rhizosphere of Radix Isatidis, which increased the indigo content by 2.3 times. Therefore, the different contents of plant secondary metabolites may be influenced by rhizosphere microorganisms directly or indirectly.

Rhizosphere soil microbial diversity is a new interdisciplinary research field in recent years, involving botany, microbiology, pedology, microbiology and ecology. It is of great significance for revealing the mechanism of plant growth and development, developing microbial resources, promoting the sustainable use of soil, regulating agricultural production and maintaining ecological service functions (Lin and Hu, 2008; Ge et al., 2019). High-throughput sequencing technology has been widely used in the study of soil microbial diversity because of its characteristics of high Qualcomm, high precision and low cost. High-throughput sequencing technology can realize the systematic study of soil microbial diversity, but there are great difficulties in the statistics and analysis of experimental data (Lou et al., 2014). Therefore, it is especially important for scientific researchers to combine microbiology, molecular biology, mathematical statistics and bioinformatics well.

## Data Availability

The original contributions presented in the study are publicly available. This data can be found here: NCBI, PRJNA1314922.
