# Supplementary material for: Environmental drivers of bacterial community diversity in the rhizosphere soil of Zanthoxylum nitidum (Roxb.) DC from different origins and their correlation with bioactive compounds
Source: Front Plant Sci. 2025 Sep 15;16:1600456. doi: 10.3389/fpls.2025.1600456 (PMC12477477; doi:10.3389/fpls.2025.1600456)
Supplement: Supplementary file 1 [file DataSheet1.docx]

Supplementary Material

# Supplementary Data

**1.1 DNA extraction and quality control**

DNA was extracted from the soil samples using a soil DNA extraction kit (Mag - Bind Soil DNA kit from OMEGA company). The extraction steps were performed following the kit's instructions. The concentration of genomic DNA extracted from each sample was determined by Qubit2.0 and loaded on 1% agarose gel to detect DNA integrity. Sample DNA meeting the requirements of concentration (0.05ng/ul~120ng/ul) and integrity was amplified by PCR.

PCR products were detected by agarose electrophoresis.The amplification products that meet the requirements of more than 400bp are recovered by magnetic bead method. The recovered DNA was accurately quantified by using Qubit3.0 DNA detection kit, and was mixed in equal amount of 1: 1 for sequencing. When mixed equally, the amount of DNA in each sample was 10ng, and the final sequencing concentration was 20pmol. The results are shown in supplementary table1.

**1.2 Quality analysis of sequencing results**

Based on the similarity level of more than 97%, we used USEARCH software (version: 5.2.236) to perform OTU clustering in the valid sequences of each sample.Then, representative otu sequences were screened out, and a total of 24607 effective OTUs were obtained.The Rarefaction Curve can be used to evaluate the quantity of sequencing data, and also to analyze species richness in samples. Therefore, the sparsity curves of six soil samples were obtained by Mothur software (version: 1.30.1) and R software (as shown in Supplementary Figure1(A)).Supplementary Figure1(A) showed that the sparse curve of each sample tends to be flat, indicating that the sequencing data of each sample is reasonable.

By plotting the relationship between the OTU number and the clustering similarity value (as depicted in Supplementary Figure1 (B)), it is evident that when the similarity reaches 97%, the curve exhibits an inflection point. Therefore, 97% is deemed the optimal clustering similarity for OTU analysis and classification. A Venn diagram of the samples can be constructed based on the sample OTUs. This Venn diagram of the six soil samples directly reflects the unique OTUs of each sample and the number of common OTUs, facilitating similarity analysis of the species composition among samples. As shown in Supplementary Figure1 (C), among the six samples, the total number of public OTUs was 173. Sample A3 had the largest number of unique OTUs, followed by sample A1 and sample B1.

**1.3 Rank-abundance Analysis**

Rank - abundance is a widely employed method for analyzing species diversity (16). It can directly reflect the richness and evenness of species within a sample. Specifically, a wider horizontal curve indicates a richer species composition, while a flatter curve shape implies a higher degree of species evenness. As shown inSupplementary Figure1(D), among the six soil samples, sample A1 had a relatively wide distribution on the horizontal axis, suggesting the most abundant species composition. In contrast, sample A2 was the narrowest on the horizontal axis, containing relatively fewer species. When evaluating based on the curve shape, the species evenness and richness presented an inverse relationship. Overall, there were slight differences in the abundance and evenness among the six samples. This indicates that the species composition and evenness of these samples were all at a satisfactory level.

# Supplementary Figures and Tables

## Supplementary Figures


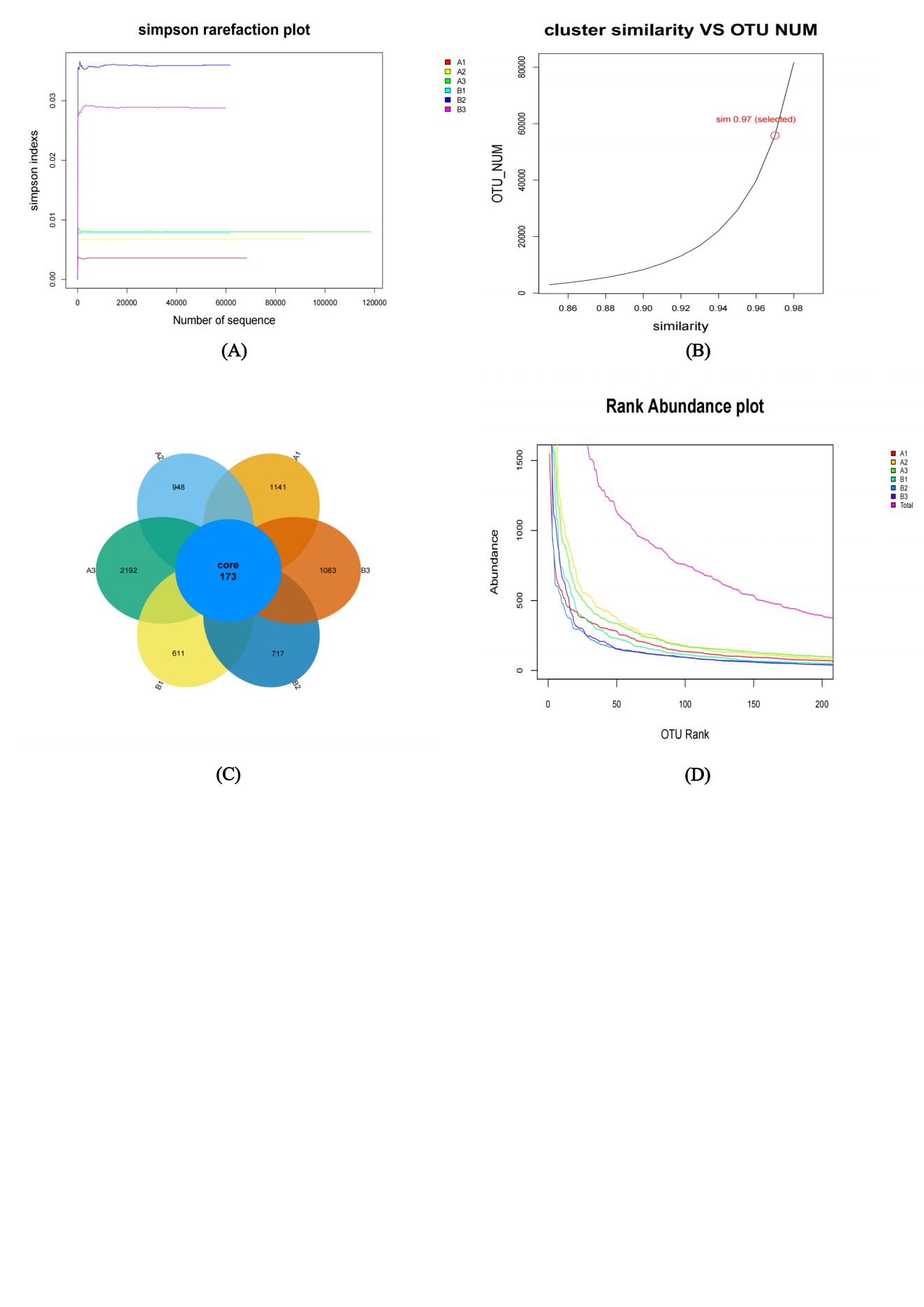


**Supplementary Figure 1.** **(A)** Rarefaction curves of 6 soil samples. Six color curves represent six samples. the shape of the curve can reflect whether the sample sequencing data amount is reasonable or not. the curve tends to be flat indicating a reasonable amount of sequencing data.**(B)** Relationship between OTU number and clustering similarity value. The relationship between different similarity values and the number of OTU is shown in the figure. A red font identifies the similarity values(0.97) used in this analysis.**(C)**Venn diagram of six soil samples. Six colors represent six samples. The overlap region represents the number of OTU common to different samples, and the non-overlapping region represents the number of unique OTU between different samples.**(D)** Rank abundance of samples species. The wider the curve on the horizontal axis is, the more abundant the species composition is. The flatter the shape of the curve, the higher the uniformity of species composition.

**2.2 Supplementary Table**

| **Supplementary Table 1. Data sheet of DNA extraction content determination** | | | |
| --- | --- | --- | --- |
| **Sample_ID** | **Sample_N** | **Sample_type** | **concentration（ng/ul）** |
| NNT-1 | A1 | soil | 34.4 |
| YLT-3 | A2 | soil | 16.4 |
| GZT-4 | A3 | soil | 24 |
| HZLMT-11 | B1 | soil | 21.2 |
| FJFZLMT-8 | B2 | soil | 37.2 |
| FJNDLMT-10 | B3 | soil | 3.5 |
